# Asperolide A, a Marine-Derived Tetranorditerpenoid, Induces G2/M Arrest in Human NCI-H460 Lung Carcinoma Cells, Is Mediated by p53-p21 Stabilization and Modulated by Ras/Raf/MEK/ERK Signaling Pathway

**DOI:** 10.3390/md11020316

**Published:** 2013-01-29

**Authors:** Cuiting Lv, Wenxia Sun, Haofen Sun, Shanjian Wei, Ruohua Chen, Bingui Wang, Caiguo Huang

**Affiliations:** 1 Department of Biochemistry and Molecular Biology, College of Basic Medical Second Military Medical University, 800 Xiangyin Road, Shanghai 200433, China; E-Mails: lvcuiting961021@126.com (C.L.); sunwenx44@yahoo.com (W.S.); sjwei8012@hotmail.com (S.W.); 2 Key Laboratory of Experimental Marine Biology, Institute of Oceanology, Chinese Academy of Sciences, Qingdao 266071, China; E-Mail: fyqfyx@163.com; 3 VIP Medicine Department, Changhai Hospital, Shanghai 200433, China

**Keywords:** asperolide A, G2/M arrest, p53-p21, Ras/Raf/MEK/ERK signaling pathway

## Abstract

Here we first demonstrate that asperolide A, a very recently reported marine-derived tetranorditerpenoid, leads to the inhibition of NCI-H460 lung carcinoma cell proliferation by G2/M arrest with the activation of the Ras/Raf/MEK/ERK signaling and p53-dependent p21 pathway. Treatment with 35 μM asperolide A (2 × IC_50_) resulted in a significant increase in the proportion of G2/M phase cells, about a 2.9-fold increase during 48 h. Immunoblot assays demonstrated time-dependent inhibition of G2/M regulatory proteins. Moreover, asperolide A significantly activated MAP kinases (ERK1/2, JNK and p38 MAP kinase) by phosphorylation, and only the inhibition of ERK activation by PD98059 reversed downregulation of G2/M regulatory proteins CDC2, and suppressed upregulation of p21 and p-p53 levels. Transfection of cells with dominant-negative Ras (RasN17) mutant genes up-regulated asperolide A-induced the decrease of cyclin B1 and CDC2, suppressed Raf, ERK activity and p53-p21 expression, and at last, abolished G2/M arrest. This study indicates that asperolide A-induced G2/M arrest in human NCI-H460 lung carcinoma cells relys on the participation of the Ras/Raf/MEK/ERK signaling pathway in p53-p21 stabilization. An *in vivo* study with asperolide A illustrated a marked inhibition of tumor growth, and little toxcity compared to Cisplatin therapy. Overall, these findings provide potential effectiveness and a theoretical basis for the therapeutic use of asperolide A in the treatment of malignancies.

## 1. Introduction

Lung cancer remains one of the leading causes of cancer-related deaths worldwide [1]. In addition, non-small-cell lung cancer (NSCLC) accounts for approximately 85% of all lung cancer cases [2]. The 5-year survival rate of non-small cell lung cancer (NSCLC) is less than 15% [3]. When feasible, surgical resection remains the single most consistent and successful option for a cure. However, chemotherapy is more beneficial for patients with advanced or metastatic disease [4]. Although systemic chemotherapy reduces the lung cancer mortality, disease progression is inevitable and dose-limiting toxicities restrict their use [5]. Therefore, conventional therapy remains largely unsuccessful and identifying new effective therapeutic treatments or novel compounds that can target cellular and molecular pathways involved in the multistep carcinogenesis process for lung cancer is urgently needed [6,7].

The Ras family, containing H-(or Ha-) Ras, K-(or Ki-) Ras, and N-Ras, which transmits extracellular signals to the interior of the cells via switching the inactive GDP-bound state to the active GTP-bound state, are commonly thought of as oncogenes; the Ras/Raf/MEK/MAPK pathway is thought to be functional downstream of EGFR (Epidermal growth-factor receptor), which has been associated with a more aggressive disease or poor prognosis in variety of cancer systems including lung cancer [8,9]. Activation of the Ras/Raf/MEK/ERK pathway or phosphorylation of ERK have been observed to increase cell death in studies of commonly used chemotherapy agents *in vitro* during the past few years, e.g., cisplatin [10], paclitaxel [11] and etoposide [12]. Furthermore, clinical trials showed that combining gemcitabine-based chemotherapy with EGFR inhibitors in NSCLC have not produced a survival advantage, and the *in vitro* findings indicated that balance between gemcitabine-induced and AG1478 (one of EGFR inhibitors)-inhibited ERK phosphorylation may have effects [13]. There are other certain circumstances under which constitutive Ras or Raf activation can lead to cell cycle arrest instead of proliferation [14]. All above suggest that the molecular mechanism of anticancer agents interacting with Ras/Raf/MEK/ERK pathway is still unclear in NSCLC, and there is a very pressing need to identify and exploit new chemotherapy for NSCLC patients.

Very recently, three new tetranorditerpenoids, asperolides A–C, were isolated and reported from a marine-derived endophytic fungus, Asperolides wenti EN-48. The results from preliminary biological evaluation demonstrated cytotoxicity of these compounds [15]. In addition, we first explored the potential anti-tumor effect of compound asperolide and wentilactone (data not shown). In the present paper, we mainly describe that the bioassay-guided fractionation of the culture extract of *Aspergillus wentii* EN-48 led to the isolation of three new tetranorlabdane diterpenoids and five related derivatives. The structures of asperolide A were herein established based on spectroscopic interpretation, and confirmed by X-ray crystallographic analysis. The absolute configuration of asperolide A was determined by application of the modified Mosher’s method.

Because of its activity against various tumor cell lines (data not shown), especially NCI-H460 cells, we test the effect of asperolide A on cell cycle progression and apoptosis in NCI-H460 cells. The results showed that there is progressive arrest in the G2-M phase, which ensures proliferation inhibition of asperolide A against NCI-H460 cells. Then we further explored the molecular mechanism of asperolide A *in vitro* and the anti-tumor effect *in vivo*. We found that *in vitro*, like the Cisplatin, it activated the Ras/Raf/MEK/ERK signaling pathway. In addition, cell cycle arrest was a p53-p21 dependent event with modulation by the Ras/Raf/MEK/ERK signaling pathway. In *in vivo* study, it was effective in inhibition of tumor xenograft growth and safer than Cisplatin in the dosage of 5 mg/kg. All of these present a potential use of asperolide A to treat NSCLC.

## 2. Results and Discussion

### 2.1. Asperolide A Inhibits the Proliferation of NCI-H460 Lung Cancer Cells

**Figure 1:**
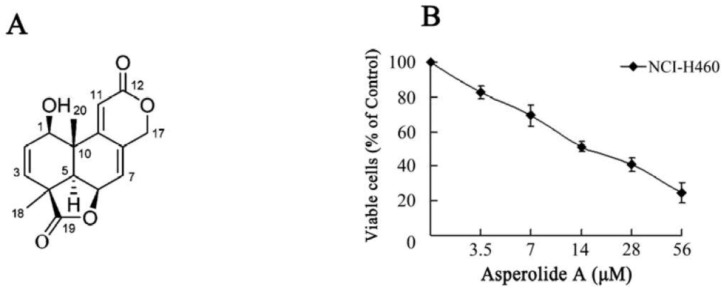
The chemical structure and cell proliferation inhibition effect of asperolide A. (**A**) Chemical structure of asperolide A; (**B**) NCI-H460 cells were treated with 3.5, 7, 14, 28 or 56 μM of asperolide A for 48h. Then, cell proliferation inhibition was analyzed by MTT assay. Values are means ±SD from three independent experiments.

The MTT assay is used to investigate the inhibitory effect of asperolide A on proliferation of NCI-H460 cells. Cells were incubated in the absence or presence of various concentrations of asperolide A (0–56 μM) for 48 h. In addition, as shown in Figure 1B, asperolide A significantly inhibited the growth of NCI-H460 cells in a dose-dependent manner. The IC_50_ value of asperolide A was 17.71 ± 3.56 μM (5.10 ± 1.02 μg/mL) for NCI-H460 cells.

### 2.2. Effect of Asperolide A on Cell Cycle Progression and Apoptosis

**Figure 2:**
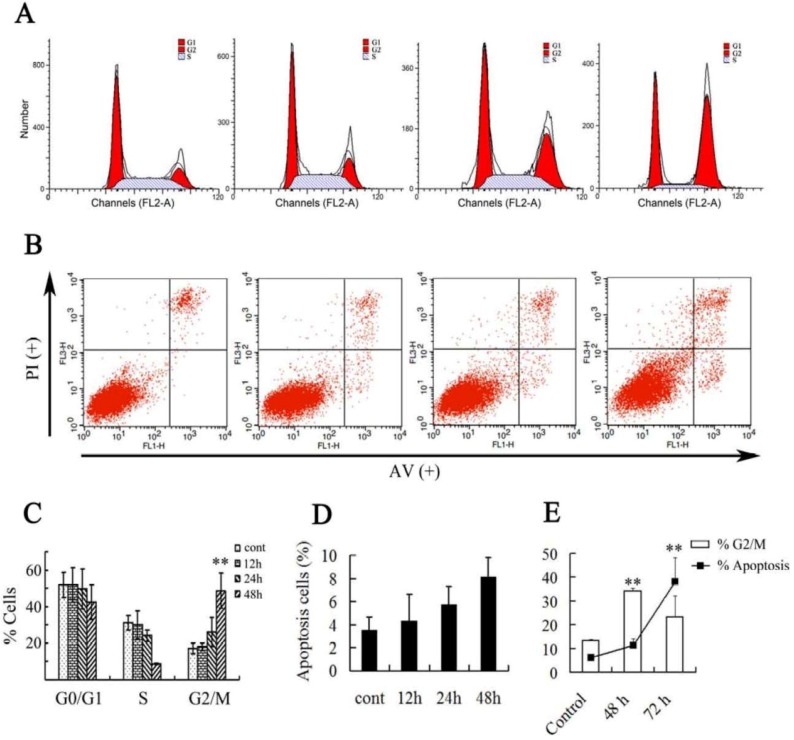
Asperolide A-induced cell cycle arrest and apoptotic cell death in NCI-H460 cells. (**A**) Asperolide A caused cell cycle arrest at the G2/M phase. Cells were treated with vehicle and 35 μM asperolide A for 12, 24, and 48 h, and cell cycle distribution was assessed by flow cytometry; (**B**) Annexin V-FITC/PI staining for apoptosis in NCI-H460 cells was assessed after 12, 24 or 48 h of treatment with 35 μM asperolide A by flow cytometry analysis; (**C**) The percentage of cells in different phases of the cell cycle was represented by a bar diagram; (**D**) The diagram showed the apoptosis rate of (**C**). (**E**) Present the change of G2/M arrest and apoptosis compared to control and 48 h treated groups after 72 h. Values were expressed as means ±SD of three independent experiments. ** *p*< 0.01 compared with control.

In order to investigate the asperolide A’s effect on proliferation, NCI-H460 cells were treated with 35 μM asperolide A (twice the IC_50_ concentration) for 12 h, 24 h and 48 h, respectively. The results showed that cells treated with asperolide A accumulated progressively in G2/M phase (Figure 2A,B). Compared with the negative control, treatment with asperolide A resulted in a significant increase in the proportion of G2/M phase cells from 24 h (control: 17.01% ± 3.03%; 24 h: 26.11% ± 7.8%; 48 h: 48.77% ± 9.58%) and about a 2.9-fold increase after 48 h. Meanwhile, G0/G1 reduced appreciably from 51.77% ± 6.97% to 42.52% ± 9.44% and the population in S phase was reduced to 8.72% ± 0.2% during 48 h incubation. These results suggest that G2/M phase arrest accounts for the antiproliferative effect of asperolide A observed in NCI-H460 cells.

Compared with cell cycle progression inhibition, asperolide A slightly triggered apoptosis in NCI-H460 cells (Figure 2C,D) in a time-dependent manner within 48 h. About 10% of cells were at apoptosis in 35 μM asperolide A-treated groups after 48 h. Therefore, we tested 72 h further. Notice that on prolonged exposure to asperolide A for 72 h, compared to 48 h, there is a dramatic increase in apoptotic cells to 38%, with concomitant deacrease in the G2/M fraction (Figure 2E). We suggest that permanent arrest in G2/M phase would make the cells more susceptible to apoptosis, and proceed programming cell death. In this paper, we will seriously discuss the effect of asperolide A on cell cycle progression.

### 2.3. ERK-p53-p21 Activation Contributes to Asperolide A-Induced Cell Cycle Arrest in NCI-H460 Cells

**Figure 3:**
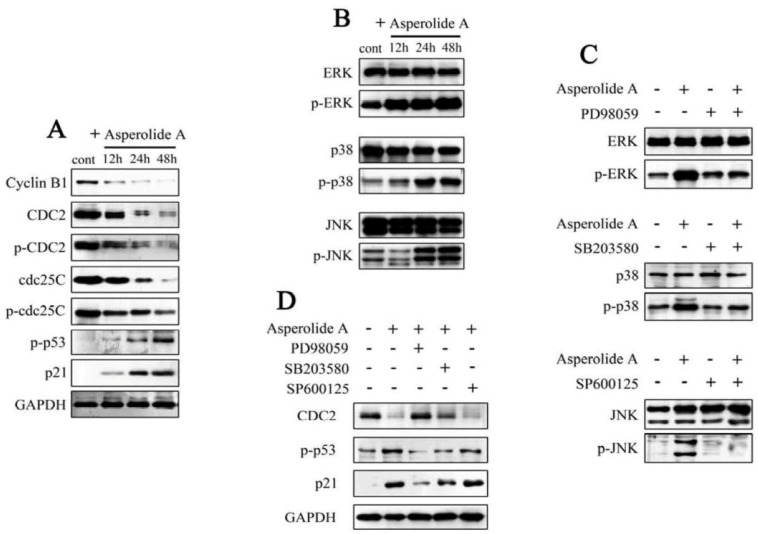
The responsibility of ERK activation for asperolide A-induced p53-p21 regulated cell cycle arrest. (**A**) NCI-H460 cells were treated with or without 35 μM asperolide A for indicated time, and then cells were harvested and lysed. cyclin B1, CDC2, p-CDC2 (Tyr15), cdc25C, p-cdc25C (Ser216), p-p53 (Ser15), p21 (Waf1/Cip1) were analyzed by Western blotting assay. GAPDH was used as an equal loading control; (**B**) Total and phosphorylated MAPK members (JNK, p38, ERK) after treatment with 35μM asperolide A for indicated time; (**C**) NCI-H460 cells were pre-treated with 20μM MEK inhibitor (PD98059), 10μM JNK inhibitor (SP600125), or 20μM P38 inhibitor (SB203580) for 2h, followed by treatment with or without 35 μM asperolide A for 48 h and the total or activation forms of MEK, JNK and p38 were evaluated by western blotting; (**D**) Cells were pre-incubated in absence or presence of MAPK inhibitors, then treated with 35 μM asperolide A, followed by immunoblotting assay performed with antibodies specific for CDC2, p-p53 and p21. Results are representative of three separate experiments. GPDH is shown as protein loading control.

To examine the mechanism responsible for asperolide A-induced cell cycle distribution, related regulatory factors were tested by using immunoblot assay. As shown in Figure 3A, asperolide A treatment of NCI-H460 cells resulted in a time-dependent decrease in the protein expression of cyclin B1, CDC2, p-CDC2, cdc25C and p-cdc25C, while increase of p-p53 and p21 expression, suggesting that changes in the expression of G2/M regulatory proteins by asperolide A are related to G2/M arrest in NCI-H460 cells. Furthermore, the elevated level of p-p53 and p21 indicate that p21 induction may be p53-dependent events and G2/M phase arrest in human NCI-H460 lung carcinoma cells is mediated by p53-p21 stabilization.

The mitogen activated protein kinase (MAPK) family contains c-Jun *N*-terminal kinase (JNK), p38 MAPK, and extra-cellular signal-regulated kinase (ERK). Over the last decade, extensive work have demonstrated that MAPK signaling pathways played critical roles in the regulation of a wide variety of cellular processes including cell growth, migration, proliferation, differentiation, development, apoptosis, and cell growth arrest [16].

To clarify whether asperolide A exerted its antitumor effects by the changes in MAPK pathway, ERK1/2, JNK and p38 MAP kinase assays were performed. The results of these experiments indicated that ERK1/2, JNK and p38 MAP kinases were significantly activated by asperolide A which increased the amount of phosphorylated ERK1/2, JNK and p38 MAP kinase (Figure 3B). To confirm the involvement of ERK, JNK and p38 in asperolide A-induced G2/M cell cycle arrest, NCI-H460 cells were pre-treated with MEK-1 inhibitor PD98059 (PD98059 is known to selectively block the activity of MEK, which activates ERK1/2 kinases), JNK inhibitor SP600125 or P38 inhibitor SB203580, followed by treatment with asperolide A. Western blotting assays demonstrated that, asperolide A-mediated phosphorylation of ERK, JNK or P38 was almost completely blocked by pre-treated ERK, JNK or P38 inhibitors as Figure 3C show, only pre-treatment with MEK-1 inhibitor PD98059 significantly blocked asperolide A-mediated activation of ERK as well as down-regulation of G2/M regulatory proteins CDC2 and suppressed up-regulation of p21 and p-p53 levels in NCI-H460 cells (Figure 3D). These results suggest that the ERK signaling pathway is required in the regulation of p53-p21-mediated G2/M-phase cell cycle arrest in response to asperolide A.

### 2.4. RasN17 Gene Transfectants Suppress the Asperolide A-Induced G2/M Arrest in NCI-H460 Cells

**Figure 4:**
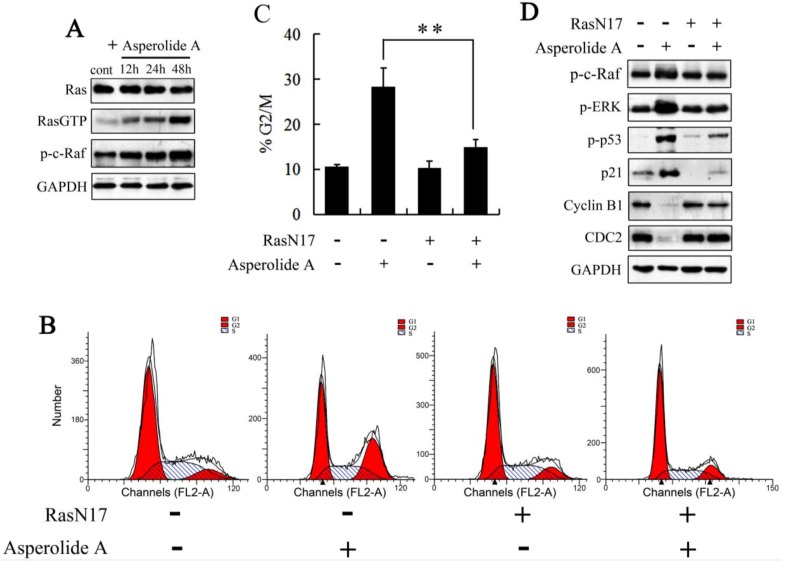
The effects of a dominant negative RasN17 mutant gene on asperolide A-induced G2/M arrest. (**A**) Ras, Ras-GTP, p-c-Raf (Ser338) were detected after asperolide A-treatment for 48h by western blotting analysis; (**B** and **C**) Cells transfected with a dominant-negative RasN17 mutant gene or not were treated with or without 35 μM asperolide A for 48 h, then harvested for cell cycle analysis or (**D**) western blotting assay against p-c-Raf, p-ERK, p-p53, p21, cyclin B1 and CDC2. GAPDH was used to ensure equal protein loading. Values were means ±SD of three independent experiments. Differences were considered statistically significant at * *p* < 0.01 when compared with asperolide A treatment in none transfected NCI-H460 cells.

As described above, the examination is essential to check whether Ras-an activator of the ERK1/2 signaling pathway-is involved in asperolide A-induced G2/M-phase arrest. Therefore, protein level of the Ras-actived form Ras-GTP was analyzed by Western blotting. As expected, asperolide A induced the activation of Ras in NCI-H460 cells, while unchanged the expression level of Ras. And c-Raf (Raf-1), the main effector recruited by GTP-bound Ras to activate the MEK-MAP kinase pathway, was also enhanced activity by phosphorylation at Ser338 (Figure 4A). Then, a dominant-negative RasN17 mutant gene was transfected into NCI-H460 cells. As shown in Figure 4B–D, cells transfected with RasN17 were not sensitive to asperolide A anymore. RasN17 gene transfectants suppressed the asperolide A-induced G2/M arrest and decrease expression of cyclin B1 and CDC2. It also blocked Raf, ERK and p53 activation, and down-regulated p21 expression.

Above all, these results suggest that the Ras/Raf/MEK/ERK signaling pathway may be involved in asperolide A-induced p53-p21-mediated G2/M-phase arrest and growth inhibition in NCI-H460 cells.

### 2.5. Asperolide A Inhibits Tumor Xenograft Growth

**Figure 5:**
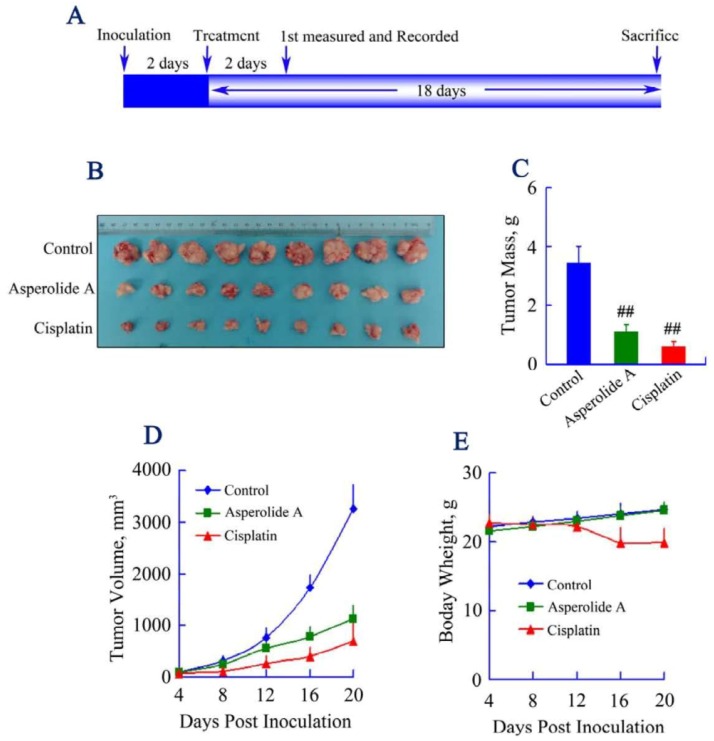
Effect of asperolide A on tumor growth in a xenograft model. (**A**) BALB/c male athymic mice were injected 5 × 10^6^ NCI-H460 cells s.c. for the development of subcutaneous tumors. The mice were randomized into 3 groups (*N* = 9) and treated with Vehicle (1% DMSO), 2.5 mg/kg Cisplatin or 5 mg/kg asperolide A i.v. according to the protocol in panel (**A**); (**B**) Tumor image from various treatment groups; (**C**) Average tumor mass at sacrifice. ## *p* < 0.001; (**D**) Tumor volume measurements; (**E**) Body weight of mice from control, Cisplatin and asperolide A treated groups.

To determine whether asperolide A inhibits tumor growth *in vivo*, equal numbers of NCI-H460 cells were injected s.c. into the right armpit of six-week old BALB/c male athymic mice. Vehicle-treated control mice (1% DMSO) or Cisplatin-treated mice (2.5 mg/kg) were used as negative or positive control group to assess effect and toxicity of asperolide A. In this study, we adopted 5 mg/kg asperolide A i.v. according to the preliminary experiments, and tumor growth inhibition was very obvious in asperolide A treated group (Figure 5B–D). Both tumor mass and volume were significantly reduced by asperolide A-treatment. The inhibition percentage of tumor growth relative to the vehicle control was 68.37%, above 50% compared to the vehicle group, but lower than that of positive control group which was 82.59%. However, as shown in Figure 5E, significant weight loss was found in the Cisplatin-treated animals. In contrast, mice given vehicle or asperolide A showed weight gain during the course of therapy.

### 2.6. Discussion

This study is the first evidence to affirm that asperolide A effectively inhibits tumor cell growth *in vitro* by induction of G2/M phase arrest, and inhibits tumor cell growth in a mouse xenograft model.

In the G2/M phase transition, it is well-known that CDC2-cyclinB1 complex is the major regulator [17]. Dephosphorylation and binding to cyclinB1 of CDC2 play essential roles in the activation of cyclinB1-CDC2 complex [18]. CDC2 is inactived by phosphorylation at Thr14 or Tyr15. cdc25C is a protein phosphatase responsible for dephosphorylating and activating CDC2 [19]. In this study, we demonstrated that asperolide A arrested the cell-cycle progression by down-regulating cyclinB and CDC2 levels. In addition, the decreased level of cdc25C by asperolide A treatment may inhibit the action of CDC2. However, on the contrary to the expectation, the protein levels of p-cdc25C (Ser216) and p-CDC2 (Tyr15) were decreased after asperolide A treatment, which might be caused by the drastic decrease in the total expression of cdc25C and CDC2.

Wt p53 is a labile protein with a short half-life. Loss of p53 function leads to genomic instability, abnormal centrosome duplication, and formation of aneuploid and polyploid cells [20,21]. Accumulation and activation of the protein can elicit cellular responses that ultimately lead to growth arrest and/or programmed cell death (apoptosis) [22,23]. The choice between arrest and cell death depends on the final integration of antagonistic signals. There is a model suggests that high levels of p53 are required for apoptosis, whereas lower levels are sufficient for growth arrest. In this study, p53 proved to be required for G2/M arrest after 48 h. Accumulation or time-dependent activation of p53 maybe account for the acceleration of apoptosis after 72 h [24,25].

p21 acts as an inhibitor of cell cycle progression [26,27]. In addition, it is one of the transcription targets of p53, which is a critical determinant in controlling both cell cycle arrest and apoptosis [28,29]. In p21-dependent G2/M phase arrest, it binds to and inhibits the activity of the cyclinB-CDC2 complex thereby causing arrest [30]. We conclude that asperolide A-induced up-regulation of p21 may be p53-dependent events and G2/M phase arrest in human NCI-H460 lung carcinoma cells is mediated by p53-p21 stabilization because (1) G2 phase arrest could be sustained only when p53 was present and capable of transcriptionally activating the cyclin-dependent kinase inhibitor p21 [31,32]; (2) p53 serine-15 phosphorylation is a pattern that is in agreement with reports that p21 is regulated by a p53-dependent mechanism in cell cycle arrest [33]; (3) Importantly, the present study found that after asperolide A administration, the level of p-p53 and p21 were all significantly elevated in a time-dependent manner (Figure 3A). Furthermore, the evidence that the induction of p-p53 and p21 is inhibited by pretreatment with the MEK inhibitors (PD98059) showed in Figure 3 supports that activation of p53-p21 is the result of the prolonged phosphorylation of ERK by asperolide A.

Ras family, which transmit extracellular signals to the interior of the cells via switching the inactive GDP-bound state to the active GTP-bound state contains H-(or Ha-) Ras, K-(or Ki-) Ras, and N-Ras. Guanylyl-imidodiphosphate (GMP-PNP) of GTP-Ras may interact specifically with c-Raf [8,34]. Raf phosphorylates MEK1/MEK2 dual-specific protein kinases, which in turn tyrosine/threonine phosphorylates extracellular-signal regulated kinase ERK1 and ERK2 of mitogen-activated protein kinases (MAPKs) [35,36]. Consider that (1) the strong and prolonged activation of ERK induced by asperolide A treatment may be caused by a positive activation of the c-Raf→MEK→ERK loop; (2) the activation of c-Raf is acquired by both Ras-dependent and independent mechanisms [37]; and (3) sustained expression of activated Ras and Raf can elicit cell-growth arrest in cancer cells *in vitro* [38], we studied on Ras, GTP-bound Ras, and p-c-Raf by western blotting assay. As expected, asperolide A induced the activation of Ras in NCI-H460 cells, while unchanged the expression level of Ras. In addition, c-Raf was also enhanced activity by phosphorylation at Ser338 (Figure 4A). So Ras→c-Raf→MEK→ERK signaling pathway may be involved in asperolide A-induced G2/M arrest in NCI-H460 cells.

In order to further explore whether the Ras/Raf/MEK/ERK pathway is required by G2/M phase cell cycle arrest induced by asperolide A in NCI-H460 cells, cells were transfected with a dominant negative Ras (RasN17) and then treated with asperolide A for 48 h. The percentage of G2/M phase cells came to normal level and asperolide A-induced increase of p-ERK, p-p53, p21 and decrease of CDC2, cyclin B1 were completely reversed by transfection with RasN17.

**Figure 6:**
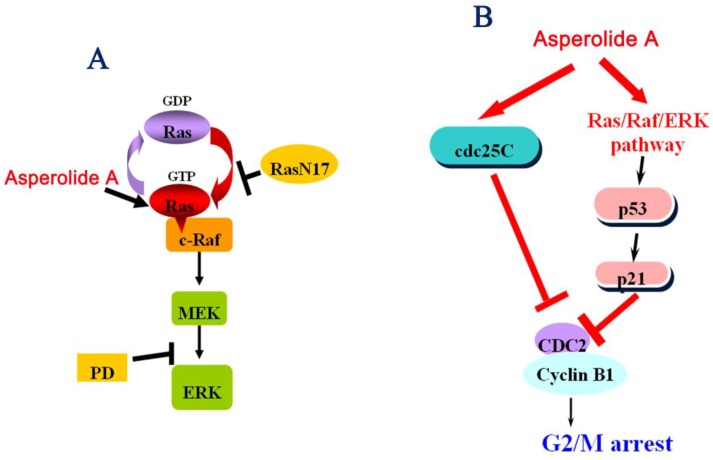
(**A**) The probable effect of asperolide A on the Ras/Raf/MEK/ERK signaling pathway; (**B**) Mechanisms of asperolide A-induced G2/M cell cycle arrest.

All above strongly suggest it is the Ras/Raf/MEK/ERK signaling pathway that is participating in p53-p21 stabilization in order to induce G2/M arrest in human NCI-H460 lung carcinoma cells. We characterize the probable effect of asperolide A on Ras/Raf/MEK/ERK signaling pathway in Figure 6A and mechanisms of asperolide A-induced G2/M cell cycle arrest in Figure 6B.

*In vitro*, cisplatin inhibits DNA synthesis by causing double-strand breaks, leading to apoptosis at the G2/M transition [39]. A link between wild-type p53 and chemotherapy-induced apoptosis shows up and cells mutated in p53 are more resistant to cisplatin than wild type cells in NSCLC cell lines [40,41]. *In vivo*, cisplatin (*cis*-diamminedichloroplatinum (II)) is a chemotherapeutic agent used in the treatment of a wide range of human malignancies, including NSCLC. Though Cisplatin combination chemotherapy is the cornerstone of treatment of many cancers, cellular drug resistance together with a narrow therapeutic range limit its use [42,43]. In addition, Cisplatin is known for its serious adverse effects, which offset the productivity of treatment and renders the chemotherapy unsustainable. Here, asperolide A-treatment significantly reduced both of the tumor mass and volume. Compared to Cisplatin, inhibition ratio was 14.22% lower. However, significant weight loss was found in the Cisplatin-treated group. That change in body weight is an important index for the initial evaluation of safety [44]; the weight gain of asperolide A-treated mice indicated the potential clinical benefit of asperolide A in reduced adverse toxicity, and the feasibility for upward adjustment of dosage for improved treatment outcomes. Therefore, toxicology and tolerant dosage study should be focused on further exploration.

## 3. Experimental Section

### 3.1. Materials

Asperolide A was isolated from a marine-derived endophytic fungus Asperolide wentii EN-48 [15]. Magnolol was purchased from Wako Pure Chemical Industries, Ltd. (Tokyo, Japan). Antibodies against cyclin B1 (#4138), CDC2 (#9112), p-CDC2 (Tyr15; #9111), cdc25C (#4688), p-cdc25C (Ser216; #9528), p-p53 (Ser15; #9284), p21 (Waf1/Cip1; #2947), p-c-Raf (Ser338; #9427), Ras (#3965), ERK (#4695), p-ERK (#4370), JNK (#9258), p-JNK (#4668), p38 (#9212) and p-p38 (#4511) were purchased from Cell Signaling Technology (Beverly, MA, USA). Ras affinity precipitation was performed with Raf-1 RBD agarose beads according to the manufacturer’s instructions (RAS activation assay kit; Upstate, Temecula, CA, USA). GAPDH was obtained from Tianjin Sungene Biotech (Tianjin, China). PD98059, SP600125 and SB203580 were obtained from Beyotime Institute of Biotechnology (Nanjing, China). The pCMV vector encoding dominant negative Ras (RasN17) was from Clontech (Mountain View, CA, USA).

### 3.2. Cell Cultures and Drug Preparation

The human large cell lung carcinoma cell line NCI-H460 was obtained from the Keygen Biotech Co., Ltd. (Nanjing, China). The cells were cultured in RPMI-1640 medium supplemented with 10% fetal bovine serum and antibiotics (100 μg/mL streptomycin and 100 U/mL penicillin). Cultures were maintained at 37 °C in an atmosphere containing 5% CO2. All the experiments were performed on logarithmically growing cells. Asperolide A was dissolved in DMSO and further diluted in PBS. The final DMSO concentration was 0.1%.

### 3.3. Cell Viability Assay

NCI-H460 cells were seeded into 96-well plates at 4 × 104 cells/mL, incubated for 24 h, and then treated with the indicated concentrations of asperolide A for 48 h. Cell viability was determined using 3-(4,5-dimeth-ylthiazol-2-yl)-2,5-diphenyltetrazolium bromide (MTT) assay.

### 3.4. Cell Apoptosis Assay

Cell apoptosis was determined by Annexin V-FITC/PI assay with Annexin V-FITC kit (Nanjing Keygen Biotech. Co., Ltd., Nanjing, China). NCI-H460 cells seeded in 6-well plates were treated with different concentrations of asperolide A for 48 h. Then, the cells were collected and re-suspended with 500 μL binding buffer at a concentration of 10^6^ cells/mL. After adding 5 μL Annexin V-FITC and 5 μL PI, cells were mixed and incubated at room temperature in the dark for 5–15 min. The samples were analyzed with a FACScalibur flow cytometer and results were calculated by CellQuest software.

### 3.5. Cell Cycle Analysis

Cells were seeded in six-well plates for 24 h and treated with asperolide A for 48 h. Then they were harvested, washed twice with PBS, fixed in 70% ethanol and stored at 4 °C overnight. Cells then were washed with PBS, incubated with RNase at 37 °C for 30 min, and then stained with PI (1 mg/mL) for at least 30 min. Cell cycle phase analysis was performed by using a FACScalibur flow cytometer. 

### 3.6. Western Blot Analysis

Cells were lysed in Western blotting lysis buffer (50 mM Tris, 150 mM NaCl, 1% Triton X-100, 1% sodium deoxycholate, 0.1% SDS and 1 mM PMSF) at 4 °C for 30 min. After 12,000× *g* centrifugation for 15 min, the protein content of supernatant was determined by BCA protein assay (Beyotime, Haimen, China). Equal amounts of the total protein samples were separated by 12% SDS-PAGE, and transferred to nitrocellulose membranes using an electro-blotting apparatus (Bio-Rad, USA). The membranes were blocked in blocking buffer (TBST plus 5% non-fat dry milk), and incubated with primary antibodies overnight at 4 °C. Then the membranes were washed with TBST and incubated with HRP (horseradish peroxidase)-conjugated secondary antibodies for 1.5 h at 4 °C. Antibody-reactive proteins were developed by the ECL system (Millipore, USA).

### 3.7. pCMV-RasN17 Vector Transfection

The pCMV vector encoding dominant negative Ras (RasN17) were purchased from Clontech (Mountain View, CA, USA). Cells were transfected with vector in 6-well plates at 5 μg final quantities per well using Xfect^TM^ Transfection Reagent (Clontech, Mountain View, CA, USA) according to the manufacturer’s instructions. Then stable transformants were selected using G418 (Calbiochem, San Diego, CA, USA). After 2 months selection, the transfected cells were used for subsequent experiments. 

### 3.8. Mouse Xenograft Model

The mouse xenograft model was established by injection of 5 × 10^6^ NCI-H460 cells s.c. into the right armpit of six-week old BALB/c male athymic mice (National Rodent Laboratory Animal Resource, Shanghai, China). The mice were randomized into vehicle control and treatment groups of nine animals when xenografts were palpable with an average size of ~100 mm^3^. Vehicle (1% DMSO) or drugs (2.5 mg/kg Cisplatin or 5 mg/kg asperolide A) was administered i.v. every other day until sacrifice. Body weight and tumor size was measured and recorded every two days from the fourth day. The tumor size was measured using electronic caliper, and tumor volumes were calculated using the formula: length × width^2^/2. 20 days post inoculation, the mice were sacrificed, the tumors collected, weighed, and photographed. The tumor inhibition effect was calculated using the following equation:

tumor suppression (%) = (1 − *T*/*C*) × 100 (1)

where *T* is the average tumor weight of the treated group and *C* is that of the control group.

### 3.9. Statistical Analysis

Data were given as means ± SD and Statistical comparisons were made by Student’s *t*-test analysis. *P*-values < 0.01 were considered to be statistically significant.

## 4. Conclusions

In summary, our data indicate that: (a) asperolide A inhibit cell-cycle progression at the G2/M phase by decreasing the levels of CDC2, cdc25c and cyclinB; (b) asperolide A-induced G2/M arrest is mediated by p53-dependent p21 induction which is regulated by Ras/Raf/MEK/ERK signaling pathway; and (c) *in vivo* studies with asperolide A showed a marked inhibition of tumor growth and little toxcity.
